# Connecting prenatal exposure to paracetamol with postnatal reproductive development: a first of its kind study in reproductive toxicology

**DOI:** 10.1093/hropen/hoag074

**Published:** 2026-09-08

**Authors:** Christian De Geyter

**Affiliations:** Reproductive Medicine and Gynecological Endocrinology (RME), University Hospital, University of Basel, Basel, Switzerland

Pregnancy is often complicated by chronic pain conditions including headaches and lower back pain. However, the use of pharmaceuticals for pain relief is challenging. Whereas opioid analgesics, including codeine, have been linked to congenital malformations and neonatal abstinence syndrome (Black *et al.*, 2019; Bowie *et al.*, 2022), most non-steroidal anti-inflammatory drugs (NSAIDs), such as ibuprofen and diclofenac, are generally not recommended during pregnancy owing to the risk of premature constriction or closure of the *ductus arteriosus* Botalli (Antonucci *et al.*, 2012; Black *et al.*, 2019). Among the NSAIDs, acetylsalicylic acid represents a notable exception as it is commonly prescribed at low dosages for the prevention of pre-eclampsia (Rolnik *et al.*, 2017).

In contrast to opioids or NSAIDs, paracetamol (acetaminophen or *N*-acetyl-*p*-amino-phenol) is widely considered as a safe medication to take during pregnancy. Owing to its perceived safety and widespread availability (AlSaeed and Elmaghraby, 2021; Maletin *et al.*, 2026) it has become the most frequently used analgesic and antipyretic pharmaceutical agent during pregnancy (Henry and Crowther, 2000). Paracetamol is well tolerated, and side effects such as nausea, vomitus and allergic reactions are rare. However, as paracetamol and its metabolites readily cross the placental barrier (Huang and Koehn, 2026), their potential effects on embryonic and foetal development have been widely discussed. The potential for paracetamol to interfere with normal neurodevelopment in offspring, including increasing the risk of autism spectrum disorders, has received considerable attention and has even elicited controversial discussions in the lay press. However, to date, the available evidence has been inconclusive (Lee *et al.*, 2026; Smith and Zipursky, 2026).

Evidence from animal studies has suggested that exposure to paracetamol during gestation affects reproductive development in both male and female offspring. Recently published experimental data indicate that prenatal exposure to high doses of paracetamol during the foetal development of male mice results in a significant increase in anogenital distance (AGD), lower testosterone but higher FSH levels, and lower sperm counts (Entezari *et al.*, 2026). In various female animal species, including mice, rat, and zebrafish, dose-dependent exposure to paracetamol during the primordial germ cell proliferation stage *in utero* leads to fewer oogonia, enhanced follicular atresia and, in several studies, reduced fertility (Holm *et al.*, 2016; Arendrup *et al.*, 2018; Moreira *et al.*, 2023; de Araújo-Ramos *et al*., 2026). In addition, experimental studies have shown that paracetamol exerts dose-dependent detrimental effects on *in vitro* cultured ovarian explants derived from first trimester (7–12 weeks of gestation) human foetuses (Lecante *et al.*, 2022): exposure to paracetamol depleted the number of germ cells in small ovarian follicles and impaired steroidogenesis, leaving the prostaglandin synthesis pathway unaffected. As such, paracetamol acts as an endocrine disruptor (Lecante *et al.*, 2022). The effects were most pronounced in ovaries taken from embryos during the 9th and 10th weeks of development (Lecante *et al.*, 2022), a period in which extensive chromatin demethylation in primordial germ cell nuclei takes place (Kawasaki *et al.*, 2014). This renders the genome of these cells particularly susceptible to changes mediated by endocrine disruptors.

Although the accumulated evidence collectively suggests a detrimental effect of paracetamol on the reproductive health of female offspring, confirmation in human populations remained lacking and was urgently needed. In this issue of *Human Reproduction Open*, Fischer *et al.* describe the COPANA study, in which the effect of intake of paracetamol during pregnancy on the ovarian and uterine morphology of the offspring was examined prospectively (Fischer *et al*., 2026). The intake of paracetamol during pregnancy, the dosage used and the medical indications were recorded through repeated online questionnaires. Exposure to paracetamol in the pregnant participants was confirmed through analysis of their urine samples. Following birth, ∼300 infant girls were assessed during mini-puberty (around 100 days of age). Ovarian size and volume, antral follicle count and uterine size were evaluated using transabdominal ultrasound. In addition, blood samples were collected to measure serum concentrations of anti-Müllerian hormone (AMH) and oestradiol (Fischer *et al.*, 2026). The data from infant girls exposed to paracetamol in either early foetal life or in mid-foetal life were then compared with those of unexposed girls. The results demonstrate that exposure to even low to moderate doses of paracetamol during early foetal development resulted in a 40% reduction of ovarian volumes with 23% fewer follicles, consistent with an observed decrease in circulating AMH levels. Secondary parameters, such as uterine volume and breast tissue diameter were also reduced, in accordance with lower circulating serum levels of oestradiol. The effects were dose-responsive, further corroborating the findings.

The COPANA study was accompanied by an independent confirmatory cohort, the Copenhagen Mother-Child Cohort, which consisted of a population-based, prospective pregnancy and birth study, in which intake of paracetamol was registered only once at the beginning of the third trimester. Exposed or non-exposed girls were examined at infancy, at puberty and at adolescence, respectively. Despite the less stringent protocol used for data collection, both ovarian and uterine volumes were found to be significantly reduced in exposed adolescent girls compared to those measured in unexposed girls. Therefore, the data of the COPANA study and the Copenhagen Mother-Child Cohort jointly point towards an impairment of both ovarian and uterine volumes in the daughters of mothers who took paracetamol during early pregnancy.

The eminent importance of Fischer *et al.*’s (2026) findings cannot be stressed enough! Ovarian volume and antral follicles as quantified during mini-puberty, during infancy and at puberty, along with lower circulating AMH levels, all suggest reduced ovarian reserve in girls exposed prenatally to paracetamol and its metabolites, especially when administered during early foetal life (until the 17th week of gestation). Whether exposure also shortens the reproductive life span of these girls in adulthood remains to be demonstrated. Furthermore, whereas the likelihood of natural conception in adult women with low circulating AMH appears uncompromised (Steiner *et al.*, 2017), the cumulative outcome of assisted reproduction, if required for other reasons, may potentially be affected in women exposed prenatally to paracetamol due to fewer oocytes being available (Polyzos *et al.*, 2018). Finally, the likelihood of early or even premature menopause may be increased in women prenatally exposed to paracetamol. Certainly, both the duration of intake and the applied dosage must be taken into consideration, this being a common principle in toxicology, but the observed dose-responsiveness of the findings points towards a distinctive effect and fits well with the already published animal research data. Recommendations on the use of paracetamol during pregnancy must now be revised (Bauer *et al.*, 2021; Smith and Zipursky, 2026).

In addition to providing novel evidence on the effects of prenatal exposure to paracetamol on ovarian reserve and uterine size, the study of Fischer *et al*. (2026) represents a major leap forward in reproductive toxicology. Until now, any links between exposure to specific medications or environmental toxicants or endocrine disruptors during pregnancy and the reproductive health of offspring were merely based on epidemiological data and have, for that reason, remained at the level of hypotheses. This has been the case with testicular dysgenesis syndrome, in which the rising incidence of hypospadias, cryptorchidism, testicular cancer and lower sperm counts was linked to the rising prevalence of toxicants and endocrine disruptors in the environment (Sharpe and Skakkebaek, 1993; Skakkebaek *et al.*, 2001; Juul *et al*., 2014). Others relied on anogenital distance as an anthropomorphic marker of prenatal exposure to oestrogens or anti-androgens (Stenz *et al.*, 2022). The present work of Fischer *et al.* (2026) directly connects accurate details on paracetamol exposure obtained prospectively during pregnancy with outcome data in the offspring during the third trimester of pregnancy, during mini-puberty and at adolescence in two independent cohorts. Its prospective design is the first of its kind and must be seen as a turning point in reproductive toxicology. Fischer *et al.*’s (2026) study is comparable to only one previous investigation of a similar topic. Unlike the present study, however, the earlier study was retrospective and described a cluster of eight very rare vaginal or cervical clear-cell adenocarcinoma cases that were observed during a short time frame (between 1946 and 1951) at a single hospital (Herbst *et al*., 1971). Retrospective interviews demonstrated that all mothers had been treated during early pregnancy with diethylstilbestrol, an oestrogenic endocrine disruptor, that was administered at that time for the prevention of miscarriage.

The insights gained from the study of Fischer *et al.* (2026) should now be built upon through long-term follow-up extending into adulthood and even up to menopause. The most formidable challenge of this task will be the rising dropout rate associated with increasingly prolonged observation periods. In the Copenhagen Mother-Child Cohort, 89.5% of girls originally included in the cohort at birth could be examined at infancy, 46.5% at puberty and only 26.2% remained available during adolescence (Fischer *et al.*, 2026). The attention this study is likely to generate among both professionals and the general public will hopefully encourage sustained long-term follow-up of the exposed individuals.
